# (*N*,*N*-Diethyl­nicotinamide-κ*N* ^1^)bis­[4,4,4-trifluoro-1-(thien-2-yl)butane-1,3-dionato-κ^2^ *O*,*O*′]copper(II)

**DOI:** 10.1107/S1600536811014401

**Published:** 2011-05-07

**Authors:** Abel M. Maharramov, Vusala I. Mardanova, Famil Chyraqov, Atash V. Gurbanov, Seik Weng Ng

**Affiliations:** a Department of Organic Chemistry, Baku State University, Baku, Azerbaijan; b Department of Chemistry, University of Malaya, 50603 Kuala Lumpur, Malaysia

## Abstract

In the title compound, [Cu(C_8_H_4_F_3_O_2_S)_2_(C_10_H_14_N_2_O)], the Cu^II^ atom exists in a distorted CuNO_4_ square-pyramidal geometry; the metal atom lies above a square plane defined by four O atoms of the two chelating anionic ligands, displaced in the direction of the axial occupant, the pyridine N atom, by 0.179 (1) Å. Weak inter­molecular C—H⋯O and C—H⋯F hydrogen bonding is present in the crystal structure. One thienyl ring is disordered over two orientations in an occupancy ratio of 0.69 (1):0.31.

## Related literature

For the related crystal structure of bis­[4,4,4-trifluoro-1-(thien-2-yl)butane-1,3-dionato]copper(II), see: Lecomte *et al.* (1988 ▶); Wang *et al.* (1996 ▶); Xu *et al.* (2010 ▶). For some adducts with *N*-heterocycles, see: Gou *et al.* (1991 ▶); Li *et al.* (1994 ▶); Liu *et al.* (1986 ▶); Yu *et al.* (1988 ▶).
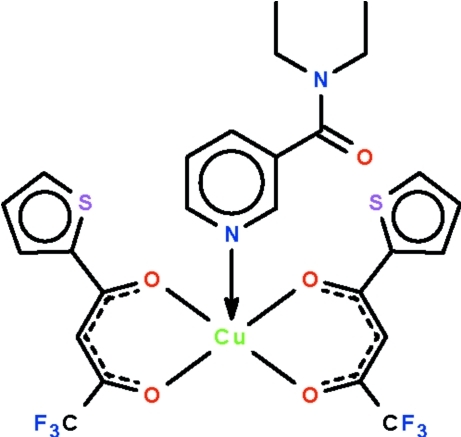


## Experimental

### 

#### Crystal data

[Cu(C_8_H_4_F_3_O_2_S)_2_(C_10_H_14_N_2_O)]
*M* *_r_* = 684.12
Triclinic, 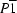

*a* = 11.4324 (5) Å
*b* = 12.8606 (5) Å
*c* = 13.0104 (5) Å
α = 62.837 (1)°
β = 64.110 (1)°
γ = 88.783 (1)°
*V* = 1492.72 (10) Å^3^
*Z* = 2
Mo *K*α radiation
μ = 0.95 mm^−1^
*T* = 293 K
0.30 × 0.30 × 0.30 mm

#### Data collection

Bruker APEXII diffractometer
Absorption correction: multi-scan (*SADABS*; Sheldrick, 1996 ▶) *T*_min_ = 0.618, *T*_max_ = 0.746
16434 measured reflections
6849 independent reflections
5423 reflections with *I* > 2σ(*I*)
*R*_int_ = 0.021

#### Refinement

*R*[*F* ^2^ > 2σ(*F* ^2^)] = 0.048
*wR*(*F* ^2^) = 0.152
*S* = 1.05
6849 reflections
392 parameters
80 restraints
H-atom parameters constrained
Δρ_max_ = 0.80 e Å^−3^
Δρ_min_ = −0.48 e Å^−3^

### 

Data collection: *APEX2* (Bruker, 2005 ▶); cell refinement: *SAINT* (Bruker, 2005 ▶); data reduction: *SAINT*; program(s) used to solve structure: *SHELXS97* (Sheldrick, 2008 ▶); program(s) used to refine structure: *SHELXL97* (Sheldrick, 2008 ▶); molecular graphics: *X-SEED* (Barbour, 2001 ▶); software used to prepare material for publication: *publCIF* (Westrip, 2010 ▶).

## Supplementary Material

Crystal structure: contains datablocks global, I. DOI: 10.1107/S1600536811014401/xu5194sup1.cif

Structure factors: contains datablocks I. DOI: 10.1107/S1600536811014401/xu5194Isup2.hkl

Additional supplementary materials:  crystallographic information; 3D view; checkCIF report

## Figures and Tables

**Table 1:** Selected bond lengths (Å)

Cu1—O1	1.9432 (19)
Cu1—O2	1.942 (2)
Cu1—O3	1.944 (2)
Cu1—O4	1.934 (2)
Cu1—N1	2.262 (2)

**Table 2:** Hydrogen-bond geometry (Å, °)

*D*—H⋯*A*	*D*—H	H⋯*A*	*D*⋯*A*	*D*—H⋯*A*
C1—H1⋯O5^i^	0.93	2.49	3.358 (7)	156
C19—H19⋯O5^ii^	0.93	2.56	3.338 (6)	141
C24—H24*C*⋯F1^iii^	0.96	2.36	3.233 (13)	151

Symmetry codes: (i) 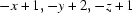; (ii) 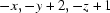; (iii) 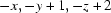.

**Fig. 1.**
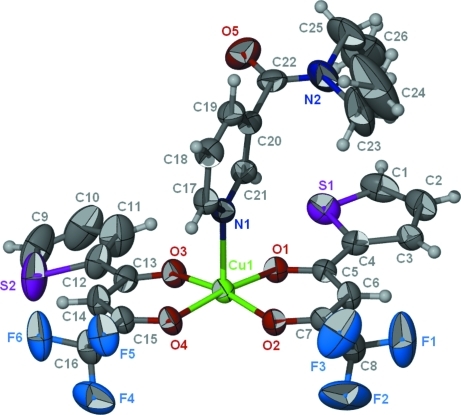
Thermal ellipsoid plot (Barbour, 2001) of Cu(C10H14N2O)(C8H4F3O2S)2 at the 50% probability level; hydrogen atoms are drawn as spheres of arbitrary radius. The disorder is not shown.

## supplementary crystallographic information

### Comment

Square-planar bis[4,4,4-trifluoro-1-(thien-2-yl)butane-1,3-dionato]copper is a Lewis acid that forms adducts with a number of *N*-heterocycles. The parent Lewis acid exists as a square-planar molecule; its crystal structure has been determined several times (Lecomte *et al.*, 1988; Wang *et al.*, 1996; Xu *et al.*, 2010). For most adducts, the Cu atom exists in a six-coordinate geometry, *e.g.*, the pyridine adduct (Liu *et al.*, 1986). The 4,4'-bipyridine adduct exists in two forms; in one form, the Cu atom is octahedrally coordinated (Gou *et al.*, 1991). The other is a dinuclear adduct in which the Cu atom shows the square-pyramidal coordination. In the title *N*,*N*-diethylbenzamide adduct (Scheme I), the Cu atom is similarly five-coordinate. The metal atom lies above the square plane defined by the O atoms of the two chelating anionic ligands in the direction of the axial occupant by 0.179 (1) Å.

### Experimental

Bis[4,4,4-trifluoro-1-(thien-2-yl)butane-1,3-dionato]copper was synthesized by using a literature procedure (Lecomte *et al.*, 1988; Wang *et al.*, 1996; Xu *et al.*, 2010). A solution of theonyltrifluoroacetylacetone (0.44 g, 0.002 mol) in ethanol (50 ml) and *N*,*N*-diethylnicotinamide (0.18 g, 0.001 mol) was added to a solution of copper sulfate pentahydrate (0.25 g, 0.001 mol) dissolved in water (50 ml). The resulting green solution has heated for a hour and then set aside for a week. The solid was filtered and recrystallized from ethanol (80%, m.p. 515 K); yield 65%. CHN&S elemental analysis. Found: C 45.69, H 3.31, S 9.48, F 16.75%; calculated for C_26_H_22_N_2_O_5_CuF_6_S_2_: C 45.61, H 3.22, S 9.36, F 16.67%.

### Refinement

Carbon-bound H-atoms were placed in calculated positions [C–H 0.93 to 0.97 Å; *U*(H) 1.2 to 1.5*U*(C)] and were included in the refinement in the riding model approximation.

One thienyl ring is disodered over two positions in a 69 (1): 31 ratio. The C–S distances were restrained to 1.70±0.01 Å and the C–C distances to 1.35±0.01 Å. The disordered rings were restrained to be nearly flat. The anisotropic temperature factors of S2 was set to those of C11', those of C9 to those of C10', those of C10 to that of C9' and those of C11 to those of S2'. The anisotropic temperature factors were restrained to be nearly isotropic.

The C–C distances of the ethyl chains were tightly restrained to 1.540±0.005 Å.

The anisotropic temperature factors of the fluorine atoms were also restrained to be nearly isotropic.

### Crystal data

**Table:**

[Cu(C_8_H_4_F_3_O_2_S)_2_(C_10_H_14_N_2_O)]	*Z* = 2
*M**_r_* = 684.12	*F*(000) = 694
Triclinic, *P*1	*D*_x_ = 1.522 Mg m^−^^3^
Hall symbol: -P 1	Mo *K*α radiation, λ = 0.71073 Å
*a* = 11.4324 (5) Å	Cell parameters from 5935 reflections
*b* = 12.8606 (5) Å	θ = 2.4–27.9°
*c* = 13.0104 (5) Å	µ = 0.95 mm^−^^1^
α = 62.837 (1)°	*T* = 293 K
β = 64.110 (1)°	Prism, green
γ = 88.783 (1)°	0.30 × 0.30 × 0.30 mm
*V* = 1492.72 (10) Å^3^	

### Data collection

**Table:**

Bruker APEXII diffractometer	6849 independent reflections
Radiation source: fine-focus sealed tube	5423 reflections with *I* > 2σ(*I*)
graphite	*R*_int_ = 0.021
φ and ω scans	θ_max_ = 27.5°, θ_min_ = 1.8°
Absorption correction: multi-scan (*SADABS*; Sheldrick, 1996)	*h* = −14→14
*T*_min_ = 0.618, *T*_max_ = 0.746	*k* = −16→16
16434 measured reflections	*l* = −16→16

### Refinement

**Table:**

Refinement on *F*^2^	Primary atom site location: structure-invariant direct methods
Least-squares matrix: full	Secondary atom site location: difference Fourier map
*R*[*F*^2^ > 2σ(*F*^2^)] = 0.048	Hydrogen site location: inferred from neighbouring sites
*wR*(*F*^2^) = 0.152	H-atom parameters constrained
*S* = 1.05	*w* = 1/[σ^2^(*F*_o_^2^) + (0.0881*P*)^2^ + 0.5656*P*] where *P* = (*F*_o_^2^ + 2*F*_c_^2^)/3
6849 reflections	(Δ/σ)_max_ = 0.001
392 parameters	Δρ_max_ = 0.80 e Å^−^^3^
80 restraints	Δρ_min_ = −0.48 e Å^−^^3^

### Fractional atomic coordinates and isotropic or equivalent isotropic displacement parameters (Å^2^)

**Table:**

	*x*	*y*	*z*	*U*_iso_*/*U*_eq_	Occ. (<1)
Cu1	0.30419 (3)	0.56562 (3)	0.41193 (3)	0.04574 (14)	
S1	0.54183 (10)	0.72016 (9)	0.54939 (11)	0.0756 (3)	
S2	0.6254 (2)	0.8905 (2)	−0.13505 (17)	0.1145 (10)	0.690 (4)
S2'	0.6483 (8)	0.9072 (7)	0.0696 (9)	0.1087 (19)	0.310 (4)
F1	0.0837 (4)	0.2134 (3)	0.8976 (3)	0.1525 (16)	
F2	0.1339 (5)	0.1709 (2)	0.7503 (4)	0.176 (2)	
F3	−0.0184 (3)	0.2524 (3)	0.7936 (4)	0.1409 (14)	
F4	0.2363 (3)	0.4454 (3)	0.1471 (4)	0.1172 (10)	
F5	0.0665 (2)	0.5072 (3)	0.2318 (2)	0.0937 (8)	
F6	0.2142 (3)	0.6186 (3)	0.0325 (2)	0.1167 (11)	
O1	0.39754 (19)	0.59008 (18)	0.4959 (2)	0.0488 (4)	
O2	0.1956 (2)	0.41288 (18)	0.5671 (2)	0.0517 (5)	
O3	0.4426 (2)	0.69749 (19)	0.2487 (2)	0.0571 (5)	
O4	0.2310 (2)	0.5291 (2)	0.3203 (2)	0.0563 (5)	
O5	0.2239 (4)	1.0114 (2)	0.4841 (4)	0.0956 (10)	
N1	0.1673 (2)	0.6845 (2)	0.4613 (2)	0.0492 (5)	
N2	0.1538 (5)	0.8623 (4)	0.6909 (4)	0.1059 (14)	
C1	0.5746 (4)	0.7210 (4)	0.6636 (5)	0.0816 (12)	
H1	0.6319	0.7840	0.6463	0.098*	
C2	0.5103 (4)	0.6226 (4)	0.7807 (5)	0.0777 (11)	
H2	0.5200	0.6105	0.8524	0.093*	
C3	0.4245 (3)	0.5360 (3)	0.7883 (4)	0.0575 (8)	
H3	0.3709	0.4635	0.8628	0.069*	
C4	0.4369 (3)	0.5821 (3)	0.6599 (3)	0.0504 (6)	
C5	0.3676 (3)	0.5306 (3)	0.6161 (3)	0.0451 (6)	
C6	0.2716 (3)	0.4211 (3)	0.7077 (3)	0.0548 (7)	
H6	0.2583	0.3806	0.7935	0.066*	
C7	0.1978 (3)	0.3724 (3)	0.6751 (3)	0.0490 (6)	
C8	0.1005 (4)	0.2514 (3)	0.7805 (3)	0.0661 (9)	
C9	0.7529 (7)	0.9912 (6)	−0.1766 (11)	0.122 (3)	0.690 (4)
H9	0.8160	1.0435	−0.2634	0.147*	0.690 (4)
C10	0.7554 (10)	0.9894 (8)	−0.0739 (10)	0.122 (3)	0.690 (4)
H10	0.8190	1.0391	−0.0801	0.146*	0.690 (4)
C11	0.6508 (12)	0.9040 (9)	0.0415 (15)	0.1087 (19)	0.690 (4)
H11	0.6366	0.8905	0.1230	0.130*	0.690 (4)
C9'	0.734 (2)	1.0246 (18)	−0.0845 (17)	0.122 (3)	0.31
H9'	0.7950	1.0882	−0.1074	0.146*	0.310 (4)
C10'	0.7052 (18)	1.0171 (19)	−0.170 (3)	0.122 (3)	0.31
H10'	0.7414	1.0720	−0.2599	0.147*	0.310 (4)
C11'	0.6135 (16)	0.9143 (16)	−0.1017 (10)	0.1145 (10)	0.31
H11'	0.5798	0.8933	−0.1447	0.137*	0.310 (4)
C12	0.5686 (4)	0.8400 (3)	0.0299 (4)	0.0743 (10)	
C13	0.4566 (3)	0.7379 (3)	0.1354 (3)	0.0562 (7)	
C14	0.3739 (4)	0.6899 (3)	0.1052 (3)	0.0648 (9)	
H14	0.3900	0.7269	0.0185	0.078*	
C15	0.2713 (3)	0.5912 (3)	0.1982 (3)	0.0556 (7)	
C16	0.1958 (4)	0.5407 (4)	0.1521 (3)	0.0730 (10)	
C17	0.0474 (3)	0.6793 (3)	0.4681 (3)	0.0534 (7)	
H17	0.0212	0.6284	0.4456	0.064*	
C18	−0.0389 (3)	0.7461 (3)	0.5069 (4)	0.0635 (8)	
H18	−0.1210	0.7415	0.5089	0.076*	
C19	−0.0020 (3)	0.8193 (3)	0.5426 (3)	0.0612 (8)	
H19	−0.0591	0.8649	0.5697	0.073*	
C20	0.1210 (3)	0.8250 (3)	0.5380 (3)	0.0523 (7)	
C21	0.2024 (3)	0.7576 (3)	0.4951 (3)	0.0523 (7)	
H21	0.2863	0.7629	0.4894	0.063*	
C22	0.1703 (4)	0.9079 (3)	0.5698 (4)	0.0650 (9)	
C23	0.0985 (10)	0.7336 (7)	0.7935 (6)	0.176 (4)	
H23A	0.1451	0.7088	0.8449	0.211*	
H23B	0.1097	0.6845	0.7526	0.211*	
C24	−0.0489 (10)	0.7181 (11)	0.8820 (10)	0.280 (9)	
H24A	−0.0870	0.6352	0.9483	0.419*	
H24B	−0.0939	0.7437	0.8301	0.419*	
H24C	−0.0589	0.7656	0.9235	0.419*	
C25	0.1991 (7)	0.9431 (6)	0.7257 (7)	0.129 (2)	
H25A	0.1360	0.9222	0.8154	0.154*	
H25B	0.2010	1.0251	0.6680	0.154*	
C26	0.3380 (8)	0.9343 (6)	0.7135 (8)	0.159 (3)	
H26A	0.3657	0.9904	0.7325	0.238*	
H26B	0.4002	0.9527	0.6255	0.238*	
H26C	0.3351	0.8545	0.7748	0.238*	

### Atomic displacement parameters (Å^2^)

**Table:**

	*U*^11^	*U*^22^	*U*^33^	*U*^12^	*U*^13^	*U*^23^
Cu1	0.0466 (2)	0.0444 (2)	0.0387 (2)	−0.00173 (14)	−0.01578 (15)	−0.01905 (15)
S1	0.0621 (5)	0.0736 (6)	0.0836 (7)	−0.0044 (4)	−0.0266 (5)	−0.0406 (5)
S2	0.1000 (14)	0.1279 (17)	0.0473 (9)	−0.0235 (11)	−0.0234 (9)	−0.0022 (9)
S2'	0.097 (2)	0.0894 (19)	0.091 (4)	−0.0344 (15)	−0.034 (2)	−0.0166 (19)
F1	0.195 (3)	0.123 (2)	0.0635 (16)	−0.084 (2)	−0.056 (2)	0.0088 (16)
F2	0.219 (4)	0.0509 (15)	0.139 (3)	−0.0227 (19)	0.008 (3)	−0.0456 (17)
F3	0.0871 (19)	0.100 (2)	0.145 (3)	−0.0385 (16)	−0.046 (2)	−0.0009 (19)
F4	0.131 (2)	0.138 (2)	0.165 (3)	0.042 (2)	−0.093 (2)	−0.116 (2)
F5	0.0675 (14)	0.127 (2)	0.0798 (15)	−0.0058 (13)	−0.0363 (12)	−0.0444 (15)
F6	0.117 (2)	0.148 (3)	0.0627 (14)	−0.0227 (18)	−0.0517 (15)	−0.0253 (15)
O1	0.0455 (10)	0.0478 (10)	0.0471 (11)	0.0010 (8)	−0.0206 (9)	−0.0203 (9)
O2	0.0570 (12)	0.0456 (10)	0.0452 (11)	−0.0042 (9)	−0.0225 (9)	−0.0186 (9)
O3	0.0571 (12)	0.0524 (11)	0.0442 (11)	−0.0072 (9)	−0.0162 (10)	−0.0178 (9)
O4	0.0608 (12)	0.0578 (12)	0.0423 (11)	−0.0058 (10)	−0.0194 (10)	−0.0234 (10)
O5	0.123 (3)	0.0534 (15)	0.117 (3)	0.0057 (16)	−0.073 (2)	−0.0324 (16)
N1	0.0512 (13)	0.0482 (13)	0.0499 (13)	0.0069 (10)	−0.0247 (11)	−0.0251 (11)
N2	0.172 (4)	0.076 (2)	0.086 (3)	0.004 (2)	−0.062 (3)	−0.051 (2)
C1	0.061 (2)	0.093 (3)	0.120 (4)	0.013 (2)	−0.044 (2)	−0.074 (3)
C2	0.077 (3)	0.098 (3)	0.094 (3)	0.021 (2)	−0.054 (2)	−0.063 (3)
C3	0.0663 (19)	0.0606 (18)	0.071 (2)	0.0141 (15)	−0.0448 (17)	−0.0397 (16)
C4	0.0415 (14)	0.0540 (16)	0.0604 (17)	0.0091 (12)	−0.0245 (13)	−0.0317 (14)
C5	0.0410 (14)	0.0476 (14)	0.0485 (15)	0.0098 (11)	−0.0203 (12)	−0.0262 (12)
C6	0.0559 (17)	0.0551 (17)	0.0452 (15)	−0.0030 (13)	−0.0221 (14)	−0.0203 (13)
C7	0.0496 (15)	0.0436 (14)	0.0449 (15)	0.0010 (12)	−0.0174 (13)	−0.0200 (12)
C8	0.075 (2)	0.0557 (18)	0.0492 (17)	−0.0144 (16)	−0.0257 (16)	−0.0150 (15)
C9	0.078 (6)	0.105 (5)	0.082 (4)	−0.013 (4)	−0.024 (5)	0.017 (4)
C10	0.090 (4)	0.079 (6)	0.121 (5)	−0.026 (4)	−0.046 (4)	0.004 (4)
C11	0.097 (2)	0.0894 (19)	0.091 (4)	−0.0344 (15)	−0.034 (2)	−0.0166 (19)
C9'	0.090 (4)	0.079 (6)	0.121 (5)	−0.026 (4)	−0.046 (4)	0.004 (4)
C10'	0.078 (6)	0.105 (5)	0.082 (4)	−0.013 (4)	−0.024 (5)	0.017 (4)
C11'	0.1000 (14)	0.1279 (17)	0.0473 (9)	−0.0235 (11)	−0.0234 (9)	−0.0022 (9)
C12	0.067 (2)	0.064 (2)	0.0507 (19)	−0.0076 (17)	−0.0194 (17)	−0.0047 (16)
C13	0.0550 (17)	0.0488 (16)	0.0433 (15)	0.0010 (13)	−0.0152 (13)	−0.0144 (13)
C14	0.069 (2)	0.064 (2)	0.0433 (16)	−0.0022 (16)	−0.0234 (15)	−0.0156 (15)
C15	0.0583 (18)	0.0597 (18)	0.0472 (16)	0.0064 (14)	−0.0244 (14)	−0.0260 (14)
C16	0.074 (2)	0.088 (3)	0.057 (2)	0.002 (2)	−0.0340 (19)	−0.0326 (19)
C17	0.0526 (16)	0.0541 (16)	0.0524 (16)	0.0036 (13)	−0.0256 (14)	−0.0249 (14)
C18	0.0512 (18)	0.068 (2)	0.069 (2)	0.0117 (15)	−0.0291 (16)	−0.0324 (18)
C19	0.0613 (19)	0.0570 (18)	0.0599 (19)	0.0201 (15)	−0.0257 (16)	−0.0287 (16)
C20	0.0638 (18)	0.0424 (14)	0.0487 (16)	0.0109 (13)	−0.0266 (14)	−0.0214 (13)
C21	0.0568 (17)	0.0479 (15)	0.0616 (18)	0.0118 (13)	−0.0347 (15)	−0.0284 (14)
C22	0.081 (2)	0.0492 (18)	0.083 (2)	0.0232 (17)	−0.047 (2)	−0.0395 (18)
C23	0.298 (12)	0.133 (5)	0.088 (4)	−0.042 (6)	−0.093 (6)	−0.045 (4)
C24	0.330 (17)	0.313 (16)	0.147 (8)	−0.124 (13)	−0.039 (9)	−0.141 (10)
C25	0.192 (7)	0.111 (4)	0.128 (5)	0.015 (4)	−0.081 (5)	−0.089 (4)
C26	0.222 (9)	0.123 (5)	0.202 (8)	0.032 (6)	−0.136 (8)	−0.099 (6)

### Geometric parameters (Å, °)

**Table:**

Cu1—O1	1.9432 (19)	C9—C10	1.339 (8)
Cu1—O2	1.942 (2)	C9—H9	0.9300
Cu1—O3	1.944 (2)	C10—C11	1.369 (10)
Cu1—O4	1.934 (2)	C10—H10	0.9300
Cu1—N1	2.262 (2)	C11—C12	1.361 (10)
S1—C1	1.688 (4)	C11—H11	0.9300
S1—C4	1.707 (3)	C9'—C10'	1.338 (11)
S2—C9	1.687 (8)	C9'—H9'	0.9300
S2—C12	1.725 (4)	C10'—C11'	1.344 (11)
S2'—C12	1.641 (9)	C10'—H10'	0.9300
S2'—C9'	1.686 (10)	C11'—C12	1.367 (10)
F1—C8	1.290 (4)	C11'—H11'	0.9300
F2—C8	1.264 (4)	C12—C13	1.463 (4)
F3—C8	1.295 (4)	C13—C14	1.414 (5)
F4—C16	1.321 (4)	C14—C15	1.369 (5)
F5—C16	1.315 (4)	C14—H14	0.9300
F6—C16	1.331 (4)	C15—C16	1.535 (5)
O1—C5	1.265 (3)	C17—C18	1.376 (5)
O2—C7	1.268 (3)	C17—H17	0.9300
O3—C13	1.252 (4)	C18—C19	1.365 (5)
O4—C15	1.262 (4)	C18—H18	0.9300
O5—C22	1.214 (5)	C19—C20	1.383 (5)
N1—C17	1.335 (4)	C19—H19	0.9300
N1—C21	1.337 (4)	C20—C21	1.373 (4)
N2—C22	1.329 (5)	C20—C22	1.502 (4)
N2—C23	1.487 (8)	C21—H21	0.9300
N2—C25	1.487 (5)	C23—C24	1.522 (5)
C1—C2	1.328 (6)	C23—H23A	0.9700
C1—H1	0.9300	C23—H23B	0.9700
C2—C3	1.440 (5)	C24—H24A	0.9600
C2—H2	0.9300	C24—H24B	0.9600
C3—C4	1.433 (5)	C24—H24C	0.9600
C3—H3	0.9300	C25—C26	1.532 (5)
C4—C5	1.467 (4)	C25—H25A	0.9700
C5—C6	1.414 (4)	C25—H25B	0.9700
C6—C7	1.365 (4)	C26—H26A	0.9600
C6—H6	0.9300	C26—H26B	0.9600
C7—C8	1.527 (4)	C26—H26C	0.9600
			
O4—Cu1—O1	171.65 (9)	C10'—C11'—H11'	119.2
O4—Cu1—O2	87.74 (9)	C12—C11'—H11'	119.2
O1—Cu1—O2	92.01 (8)	C11—C12—C13	127.8 (7)
O4—Cu1—O3	92.32 (9)	C11'—C12—C13	134.9 (10)
O1—Cu1—O3	86.06 (9)	C11'—C12—S2'	105.0 (10)
O2—Cu1—O3	167.13 (10)	C13—C12—S2'	118.7 (4)
O4—Cu1—N1	97.55 (9)	C11—C12—S2	108.8 (7)
O1—Cu1—N1	90.74 (8)	C13—C12—S2	123.2 (3)
O2—Cu1—N1	98.28 (9)	S2'—C12—S2	118.1 (4)
O3—Cu1—N1	94.47 (9)	O3—C13—C14	124.4 (3)
C1—S1—C4	91.7 (2)	O3—C13—C12	115.9 (3)
C9—S2—C12	90.7 (4)	C14—C13—C12	119.8 (3)
C12—S2'—C9'	94.1 (12)	C15—C14—C13	122.7 (3)
C5—O1—Cu1	127.36 (18)	C15—C14—H14	118.6
C7—O2—Cu1	123.57 (18)	C13—C14—H14	118.6
C13—O3—Cu1	127.3 (2)	O4—C15—C14	129.2 (3)
C15—O4—Cu1	123.9 (2)	O4—C15—C16	112.7 (3)
C17—N1—C21	117.6 (3)	C14—C15—C16	118.1 (3)
C17—N1—Cu1	123.2 (2)	F5—C16—F4	106.9 (3)
C21—N1—Cu1	119.0 (2)	F5—C16—F6	106.9 (3)
C22—N2—C23	124.4 (4)	F4—C16—F6	106.7 (3)
C22—N2—C25	118.5 (4)	F5—C16—C15	112.5 (3)
C23—N2—C25	117.1 (4)	F4—C16—C15	110.5 (3)
C2—C1—S1	113.3 (3)	F6—C16—C15	113.1 (3)
C2—C1—H1	123.4	N1—C17—C18	122.8 (3)
S1—C1—H1	123.4	N1—C17—H17	118.6
C1—C2—C3	115.0 (4)	C18—C17—H17	118.6
C1—C2—H2	122.5	C19—C18—C17	118.9 (3)
C3—C2—H2	122.5	C19—C18—H18	120.6
C4—C3—C2	107.4 (3)	C17—C18—H18	120.6
C4—C3—H3	126.3	C18—C19—C20	119.4 (3)
C2—C3—H3	126.3	C18—C19—H19	120.3
C3—C4—C5	129.2 (3)	C20—C19—H19	120.3
C3—C4—S1	112.5 (2)	C21—C20—C19	118.1 (3)
C5—C4—S1	118.2 (2)	C21—C20—C22	119.8 (3)
O1—C5—C6	123.8 (3)	C19—C20—C22	122.1 (3)
O1—C5—C4	116.4 (3)	N1—C21—C20	123.3 (3)
C6—C5—C4	119.7 (3)	N1—C21—H21	118.4
C7—C6—C5	122.5 (3)	C20—C21—H21	118.4
C7—C6—H6	118.7	O5—C22—N2	123.7 (4)
C5—C6—H6	118.7	O5—C22—C20	118.8 (3)
O2—C7—C6	129.7 (3)	N2—C22—C20	117.4 (3)
O2—C7—C8	112.2 (2)	N2—C23—C24	108.1 (8)
C6—C7—C8	118.1 (3)	N2—C23—H23A	110.1
F2—C8—F3	103.8 (4)	C24—C23—H23A	110.1
F2—C8—F1	108.3 (4)	N2—C23—H23B	110.1
F3—C8—F1	104.5 (4)	C24—C23—H23B	110.1
F2—C8—C7	111.5 (3)	H23A—C23—H23B	108.4
F3—C8—C7	112.6 (3)	C23—C24—H24A	109.5
F1—C8—C7	115.2 (3)	C23—C24—H24B	109.5
C10—C9—S2	114.3 (8)	H24A—C24—H24B	109.5
C10—C9—H9	122.8	C23—C24—H24C	109.5
S2—C9—H9	122.8	H24A—C24—H24C	109.5
C9—C10—C11	110.2 (12)	H24B—C24—H24C	109.5
C9—C10—H10	124.9	N2—C25—C26	111.8 (5)
C11—C10—H10	124.9	N2—C25—H25A	109.3
C12—C11—C10	116.1 (12)	C26—C25—H25A	109.3
C12—C11—H11	122.0	N2—C25—H25B	109.3
C10—C11—H11	122.0	C26—C25—H25B	109.3
C10'—C9'—S2'	113 (2)	H25A—C25—H25B	107.9
C10'—C9'—H9'	123.4	C25—C26—H26A	109.5
S2'—C9'—H9'	123.4	C25—C26—H26B	109.5
C9'—C10'—C11'	106 (2)	H26A—C26—H26B	109.5
C9'—C10'—H10'	126.9	C25—C26—H26C	109.5
C11'—C10'—H10'	126.9	H26A—C26—H26C	109.5
C10'—C11'—C12	122 (2)	H26B—C26—H26C	109.5
			
O2—Cu1—O1—C5	−11.0 (2)	C10'—C11'—C12—C11	−3.7 (9)
O3—Cu1—O1—C5	−178.3 (2)	C10'—C11'—C12—C13	166.2 (9)
N1—Cu1—O1—C5	87.3 (2)	C10'—C11'—C12—S2'	0.7 (6)
O4—Cu1—O2—C7	177.2 (2)	C10'—C11'—C12—S2	−137 (3)
O1—Cu1—O2—C7	5.6 (2)	C9'—S2'—C12—C11	27 (4)
O3—Cu1—O2—C7	86.7 (4)	C9'—S2'—C12—C11'	−0.4 (4)
N1—Cu1—O2—C7	−85.5 (2)	C9'—S2'—C12—C13	−168.8 (7)
O4—Cu1—O3—C13	5.1 (3)	C9'—S2'—C12—S2	13.4 (6)
O1—Cu1—O3—C13	176.9 (3)	C9—S2—C12—C11	0.3 (4)
O2—Cu1—O3—C13	95.1 (5)	C9—S2—C12—C11'	50 (2)
N1—Cu1—O3—C13	−92.7 (3)	C9—S2—C12—C13	−175.1 (4)
O2—Cu1—O4—C15	−172.3 (3)	C9—S2—C12—S2'	2.6 (5)
O3—Cu1—O4—C15	−5.2 (3)	Cu1—O3—C13—C14	−2.5 (5)
N1—Cu1—O4—C15	89.6 (3)	Cu1—O3—C13—C12	178.7 (2)
O4—Cu1—N1—C17	23.0 (2)	C11—C12—C13—O3	−7.5 (6)
O1—Cu1—N1—C17	−158.0 (2)	C11'—C12—C13—O3	−174.7 (9)
O2—Cu1—N1—C17	−65.8 (2)	S2'—C12—C13—O3	−10.7 (6)
O3—Cu1—N1—C17	115.9 (2)	S2—C12—C13—O3	167.0 (3)
O4—Cu1—N1—C21	−162.3 (2)	C11—C12—C13—C14	173.7 (5)
O1—Cu1—N1—C21	16.8 (2)	C11'—C12—C13—C14	6.5 (10)
O2—Cu1—N1—C21	108.9 (2)	S2'—C12—C13—C14	170.5 (5)
O3—Cu1—N1—C21	−69.3 (2)	S2—C12—C13—C14	−11.8 (5)
C4—S1—C1—C2	−0.1 (3)	O3—C13—C14—C15	−1.8 (6)
S1—C1—C2—C3	−0.9 (5)	C12—C13—C14—C15	176.9 (4)
C1—C2—C3—C4	1.6 (5)	Cu1—O4—C15—C14	3.2 (5)
C2—C3—C4—C5	−177.9 (3)	Cu1—O4—C15—C16	179.6 (2)
C2—C3—C4—S1	−1.6 (4)	C13—C14—C15—O4	1.4 (6)
C1—S1—C4—C3	1.0 (3)	C13—C14—C15—C16	−174.9 (3)
C1—S1—C4—C5	177.7 (3)	O4—C15—C16—F5	43.8 (4)
Cu1—O1—C5—C6	11.2 (4)	C14—C15—C16—F5	−139.3 (4)
Cu1—O1—C5—C4	−167.59 (18)	O4—C15—C16—F4	−75.6 (4)
C3—C4—C5—O1	−179.8 (3)	C14—C15—C16—F4	101.3 (4)
S1—C4—C5—O1	4.1 (4)	O4—C15—C16—F6	165.0 (3)
C3—C4—C5—C6	1.3 (5)	C14—C15—C16—F6	−18.1 (5)
S1—C4—C5—C6	−174.7 (2)	C21—N1—C17—C18	0.6 (5)
O1—C5—C6—C7	−3.1 (5)	Cu1—N1—C17—C18	175.4 (2)
C4—C5—C6—C7	175.7 (3)	N1—C17—C18—C19	−1.3 (5)
Cu1—O2—C7—C6	−0.4 (5)	C17—C18—C19—C20	0.4 (5)
Cu1—O2—C7—C8	179.1 (2)	C18—C19—C20—C21	1.1 (5)
C5—C6—C7—O2	−2.7 (5)	C18—C19—C20—C22	177.1 (3)
C5—C6—C7—C8	177.8 (3)	C17—N1—C21—C20	1.1 (5)
O2—C7—C8—F2	65.6 (5)	Cu1—N1—C21—C20	−174.0 (2)
C6—C7—C8—F2	−114.9 (4)	C19—C20—C21—N1	−1.9 (5)
O2—C7—C8—F3	−50.6 (4)	C22—C20—C21—N1	−178.0 (3)
C6—C7—C8—F3	128.9 (4)	C23—N2—C22—O5	−174.8 (6)
O2—C7—C8—F1	−170.4 (4)	C25—N2—C22—O5	2.2 (7)
C6—C7—C8—F1	9.1 (5)	C23—N2—C22—C20	4.3 (8)
C12—S2—C9—C10	−0.12 (18)	C25—N2—C22—C20	−178.7 (5)
S2—C9—C10—C11	−0.1 (2)	C21—C20—C22—O5	92.2 (4)
C9—C10—C11—C12	0.3 (5)	C19—C20—C22—O5	−83.7 (5)
C12—S2'—C9'—C10'	0.14 (19)	C21—C20—C22—N2	−86.9 (5)
S2'—C9'—C10'—C11'	0.2 (2)	C19—C20—C22—N2	97.2 (5)
C9'—C10'—C11'—C12	−0.6 (5)	C22—N2—C23—C24	−97.6 (7)
C10—C11—C12—C11'	−14.3 (9)	C25—N2—C23—C24	85.4 (7)
C10—C11—C12—C13	174.7 (5)	C22—N2—C25—C26	−97.1 (7)
C10—C11—C12—S2'	−168 (4)	C23—N2—C25—C26	80.1 (8)
C10—C11—C12—S2	−0.4 (6)		

### Hydrogen-bond geometry (Å, °)

**Table:**

*D*—H···*A*	*D*—H	H···*A*	*D*···*A*	*D*—H···*A*
C1—H1···O5^i^	0.93	2.49	3.358 (7)	156
C19—H19···O5^ii^	0.93	2.56	3.338 (6)	141
C24—H24C···F1^iii^	0.96	2.36	3.233 (13)	151

Symmetry codes: (i) −*x*+1, −*y*+2, −*z*+1; (ii) −*x*, −*y*+2, −*z*+1; (iii) −*x*, −*y*+1, −*z*+2.
